# The PACE trial of treatments for chronic fatigue syndrome: a response to WILSHIRE et al

**DOI:** 10.1186/s40359-019-0288-x

**Published:** 2019-03-12

**Authors:** Michael Sharpe, Kim Goldsmith, Trudie Chalder

**Affiliations:** 1 University of Oxford Department of Psychiatry, Warneford Hospital, Oxford, OX3 7JX UK; 2 Biostatistics & Health Informatics Department, Division of Psychology and Systems Sciences, Institute of Psychiatry, Psychology & Neuroscience, King’s College London, London, UK; 3 Academic Department of Psychological Medicine, King’s College London, London, UK

**Keywords:** Clinical trial, Chronic fatigue syndrome, Methodology, Cognitive behaviour therapy, Graded exercise therapy

## Abstract

Chronic Fatigue Syndrome (CFS) is chronic disabling illness characterized by severe disabling fatigue, typically made worse by exertion. Myalgic Encephalomyelitis (ME) is thought by some to be the same disorder (then referred to as CFS/ME) and by others to be different. There is an urgent need to find effective treatments for CFS. The UK Medical Research Council PACE trial published in 2011 compared available treatments and concluded that when added to specialist medical care, cognitive behaviour therapy and graded exercise therapy were more effective in improving both fatigue and physical function in participants with CFS, than both adaptive pacing therapy and specialised medical care alone. In this paper, we respond to the methodological criticisms of the trial and a reanalysis of the trial data reported by Wilshire at al. We conclude that neither the criticisms nor the reanalysis offer any convincing reason to change the conclusions of the PACE trial.

## The PACE trial

Chronic Fatigue Syndrome (CFS) is a chronic disabling illness characterized by severe disabling fatigue, typically made worse by exertion. Myalgic Encephalomyelitis (ME) is thought by some to be the same disorder (then referred to as CFS/ME) and by others to be different. The cause of CFS is unknown. There is an urgent need for effective treatments.

The PACE trial compared the effectiveness of available non-drug treatments. In a four-arm randomised trial it compared three therapies, given as additions to specialist medical care (SMC) and SMC alone. It found that, 12 months after randomisation, two of the therapies, cognitive behaviour therapy (CBT) and graded exercise therapy (GET) were more effective in improving both patient-reported fatigue and physical functioning (the main defining symptoms of CFS), than either adaptive pacing therapy (APT) or specialist medical care (SMC) alone [1].

The trial was funded by the UK Medical Research Council (MRC). Six hundred forty-one participants were recruited after rigorous assessment for eligibility from six clinical services in the UK; randomisation was done by an independent Trials Unit; the trial treatments were specified in detailed manuals and were rigorously monitored for quality; there was very little loss to follow up (5 %) at the final 12 month outcome assessment; the main trial analysis was conducted by a statistician (KG) blind to treatment allocation and supervised by an additional and senior MRC statistician. Whilst, as with almost all behavioural medicine trials, it was not possible to blind participants to the treatment allocation, the delivery of the three therapies was carefully matched for patient contact time to mimimise non-specific treatment effects. Finally, all aspects of the trial were overseen by an MRC approved independent Trial Steering Committee and Data Monitoring Committee. The trial was registered (ISRCTN54285094) and both the protocol and a detailed statistical analysis plan (SAP) were published [2, 3]. The transparency of the PACE trial procedures was recognised in a review by the UK Health Research Authority (HRA) [4].

The main trial findings were published in the Lancet in 2011. A number of secondary papers were published subsequently. One secondary paper was an exploration of the relative rates of ‘recovery’ from CFS with each of the trial treatments which reported that recovery was more likely with CBT and GET than with the other two treatments [5]. Another secondary paper was a post-trial long-term naturalistic follow-up of trial participants which found that the benefits of CBT and GET were maintained 18 months after the end of the trial [6].

Here we respond to the recent paper by Wilshire et al. which is a combined critique of all of the three trial papers mentioned above [7]. We address each of the main points they make in turn:

## The PACE trial analysis plan

Wilshire et al. say that changes from an initial outline analysis plan described in the trial protocol published in 2007 [2] to the final approved statistical analysis plan (SAP) published in 2013 [3] were ‘highly problematic’ and led to bias in the trial outcomes.

We disagree that these changes were either ‘problematic’ or a source of bias. There was no change in the designated primary outcomes. The changes, which were only in the scoring of the pre-specified outcomes, were made in response to statistical advice, approved by the Trial Data Monitoring and Steering Committees and made before the data was analysed, as confirmed by a UK HRA review of the trial conduct [4]. They were also documented in the relevant published papers [1]. The changes made were: (a) to use mean scores for each primary outcome (fatigue and physical function) separately rather than as a composite outcome because composite outcomes are difficult to interpret [8]. (b) to use Likert type scoring (0, 1, 2, 3) rather binary scoring (0, 0, 1, 1) for the fatigue scale to make the measure more accurate and sensitive to change.

## Reanalysis of the PACE trial data

Wilshire et al. report that, contrary to our findings, their reanalysis of the trial data, which they based on our preliminary analysis plan, yielded ‘null outcomes’.

We have read their reanalysis carefully and find it unconvincing. This is because: First, it assumes, without justification, that the original proposed scoring of primary outcomes described above, was more valid than the method described in the final approved analysis plan. Second, their reanalysis appears not to have been based on a clear a priori analysis plan. Third, it only used part of the trial dataset. Fourth, it employed analytic strategies we consider questionable: For example, they omitted data from an entire treatment arm of the four-arm trial (APT). Despite these differences from our published analysis, Wilshire et al. still found that both CBT and GET were statistically superior to their only comparison treatment (SMC, which they choose to refer to as ‘control’). However, they then abolished this statistical significance by applying an excessive Bonferroni correction for multiple testing (multiplying the required *p* values by five or six, rather than by a more appropriate two) [7]. We have also previously published an analysis of the trial based on the originally proposed but superseded scoring methods favoured by Wilshire et al. and found that the relative effect of the treatments remained similar to that reported in the published PACE trial paper (with only one of the planned comparisons was no longer statistically significant) [9, 10].

## Patient reported outcomes

Wilshire et al. suggest that, as the primary outcomes in PACE were participant-rated (CFS is defined by self-report as there are no reliable objective measures) and the participants inevitably knew what treatment they were given (the therapies tested could not be blinded as they were collaborative between therapist and participant), the trial findings can be dismissed as simply due to participant bias when rating outcomes.

We disagree with this proposition. Whilst participant rated outcomes do potentially pose a risk of bias for all trials testing the effect of unblindable treatments, we do not agree that this is a convincing alternative explanation for the PACE trial findings. This is because: First, participants did not just give global ratings at the end of treatment, they answered specific questions about fatigue and function, as long as six months after therapy was completed, making a transient ‘placebo’ type effect very unlikely. Second, the majority of the trial secondary measures showed a similar pattern as the primary outcomes. Third, and most importantly, the trial design controlled for a non-specific effect of treatment by comparing three therapies (CBT, GET and APT) that were all attention and credibility matched. Credibility matching was determined by asking participants how logical they found the treatment they were allocated to and how confident they were it would help them before they received it; the credibility rating of APT was higher than CBT, and similar to GET. Despite this control the biggest difference in outcomes was between APT and both CBT and GET. Indeed, one could argue that if the participant rated outcomes had been biased by the non-specific factors, such as perceived credibility, APT should have performed best, when in fact it performed worst.

## Recovery

The issue of recovery from CFS was considered in a secondary PACE paper which sought to estimate relative proportions of participants in each trial arm who might be considered to have ‘recovered’ from CFS [5]. We reported that the rates of recovery, whilst modest, were greater with CBT and GET than with APT and SMC alone. Wilshire et al. report that their reanalysis of PACE data yielded no difference in the rates of recovery between trial arms.

Whilst we agree that there is no generally accepted definition of recovery from CFS, we disagree with this conclusion [11]. In our paper we explored a number of definitions of recovery using various thresholds on a number of trial outcome variables. These were specified before doing the analysis. Different definitions produced different absolute rates of recovery, but a similar relative difference between treatments, always favouring CBT and GET [5, 12]. Wilshire et al. did a single analysis using only our proposed but superseded criteria for recovery, which specified very high thresholds on the outcome scales. They found only a small proportion (between three and 7 %) of patients could be considered ‘recovered’ in any of the trial arms. Inevitably, these very small absolute differences between treatments became statistically non-significant [7].

We prefer the definitions of recovery we used to those used by Wilshire et al. as they give absolute rates more consistent both with the literature, and with our clinical experience. We also note that, even in Wilshire et al.’s analysis, the relative rate of recovery with CBT and GET was approximately twice that with APT and SMC alone.

## Persistence of treatment effects

We examined long term (mean of 2.5 years from trial entry) outcomes of trial treatment in a further paper [6]. Wilshire et al. dispute our finding that the benefit of CBT and GET seen in both fatigue and physical function at 12 months was maintained in the long-term.

We disagree with their conclusion which they base on an analysis that compared the long-term outcomes *between* the originally randomised treatment groups [7]. As we explained in the paper, following the final trial 12-month outcome, patients were no longer in the trial. That is, for the subsequent 18 months of the long-term follow up we reported, treatment was no longer given according to the original random allocation. Indeed, as part of our original promise to participants, those who remained unwell at the trial final follow up were offered additional treatments by PACE trial therapists. The type of additional treatment was determined by the participant’s SMC doctor, in negotiation with the participant. It turned out that many participants allocated to APT and SMC in the trial, received CBT or GET during the post-trial follow up period. Consequently, it is misleading to analyse the data as a comparison between originally randomised groups as the random allocation no longer applies and analysing data on a subgroup does not overcome the problem of confounding of outcome and treatment given.

For these reasons we reported our main analysis as a comparison of patient outcomes over time *within, not between,* their originally allocated groups. The graphs in Fig. 2 and the figures in Table 3 of our follow up paper, show clearly that the improvements in mean fatigue and physical function scores found at the end of the trial in participants originally allocated to CBT and GET, were maintained at long-term follow up [6]. They also show, as we reported, that patients who were allocated to APT and SMC in the trial, many of whom had received additional CBT of GET after the trial, also improved over the post-trial follow up period. In summary the improvements in fatigue and functioning seen from baseline to final trial outcome in those originally allocated to CBT and GET, were maintained at long-term post-trial follow up.

## Replication of the PACE trial findings

The ultimate test of any scientific finding is replication. The PACE trial replicated findings from many earlier randomised trials; systematic reviews and meta-analyses support the efficacy of both CBT and GET as treatments for CFS [13–17]. Furthermore, trials published after PACE continue to find that these treatments to be both safe and moderately effective for CFS and for related syndromes [18–22]. Clinical case series continue to support their translation into clinical practice [22] and in a qualitative study the majority of those who had been given CBT reported that they were satisfied with it [23]. In summary, the findings of PACE do not stand alone but have been replicated many times. These multiple replications make the findings likely to be robust.

## Other comments on the PACE trial findings

One often repeated reason for rejecting the PACE trial findings, mentioned by Wilshire et al., is that the benefit of rehabilitative therapies, especially GET found in trials, is not reported by some patients in surveys [7]. This discrepancy between the finding of clinical trials and those of patient group surveys certainly requires explanation: One explanation may be the patients who respond to surveys are dissimilar to those who participated in the trial. Another possible explanation may be the treatments given outside of a trial were not as well delivered, for example by being insufficiently collaborative or recommending too rapid increases in activity. We suggest that further research is needed to understand this apparent difference between the finding of clinical trials and those of patient surveys.

Another  argument alluded to by Wilshire et al. for rejecting the findings of the PACE trial is that they imply that CFS or ME is psychiatric or psychological in nature (an illness designation still strongly stigmatised in our society) rather than purely biomedical [24]. However understandable this view may be, we believe it to be mistaken. The evidence that CBT and GET help patients with CFS does not in fact indicate the cause of their illness. Indeed, the PACE trial main paper explicitly states: “*The effectiveness of behavioural treatments does not imply that the condition is psychological in nature*” [1]. In this context, it is also worth noting that similar therapies have also been found to improve fatigue in illnesses with established physical pathology such as multiple sclerosis [25].

## Conclusions

PACE was a large carefully designed and intensively monitored clinical trial of different non-drug treatments for CFS. Like all trials, it had limitations that are clearly described in the papers reporting it. However, after carefully reviewing Wilshire et al’s criticisms of the PACE trial findings, we can find no good reason to change its conclusions.

We therefore restate that the PACE trial found that both CBT and GET, when given appropriately as supplements to specialist medical care, are more effective in improving both fatigue and physical functioning in people with CFS, than are APT and SMC alone.

## Abbreviations

APT: Adaptive Pacing Therapy
CBT: Cognitive Behaviour Therapy
CFS: Chronic Fatigue Syndrome
GET: Graded ExerciseTherapy
ME: Myalgic Encephalomyelitis
MRC: Medical Research Council of the United Kingdom
PACE: A comparison of adaptive Pacing therapy, Cognitive behaviour therapy, graded exercise therapy, and specialist medical care for chronic fatigue syndrome: a randomised Evaluation
SMC: Specialist Medical Care
UK HRA: UK Health Research Authority
